# Hydrogel-Based Nanocomposites and Mesenchymal Stem Cells: A Promising Synergistic Strategy for Neurodegenerative Disorders Therapy

**DOI:** 10.1155/2013/270260

**Published:** 2013-12-26

**Authors:** Diego Albani, Antonio Gloria, Carmen Giordano, Serena Rodilossi, Teresa Russo, Ugo D'Amora, Marta Tunesi, Alberto Cigada, Luigi Ambrosio, Gianluigi Forloni

**Affiliations:** ^1^IRCCS-“Mario Negri” Institute for Pharmacological Research, Via La Masa 19, 20156 Milan, Italy; ^2^Institute of Composite and Biomedical Materials, National Research Council of Italy, P.le Tecchio 80, 80125 Naples, Italy; ^3^Department of Chemistry, Materials and Chemical Engineering “G. Natta,” Politecnico of Milan and INSTM Consortium Research Unit, 20131 Milan, Italy

## Abstract

Hydrogel-based materials are widely employed in the biomedical field. With regard to central nervous system (CNS) neurodegenerative disorders, the design of injectable nanocomposite hydrogels for *in situ* drug or cell release represents an interesting and minimally invasive solution that might play a key role in the development of successful treatments. In particular, biocompatible and biodegradable hydrogels can be designed as specific injectable tools and loaded with nanoparticles (NPs), to improve and to tailor their viscoelastic properties upon injection and release profile. An intriguing application is hydrogel loading with mesenchymal stem cells (MSCs) that are a very promising therapeutic tool for neurodegenerative or traumatic disorders of the CNS. This multidisciplinary review will focus on the basic concepts to design acellular and cell-loaded materials with specific and tunable rheological and functional properties. The use of hydrogel-based nanocomposites and mesenchymal stem cells as a synergistic strategy for nervous tissue applications will be then discussed.

## 1. Introduction

Mesenchymal stem cells (MSCs) are a very promising therapeutic tool for neurodegenerative or traumatic disorders affecting the central nervous system (CNS). For instance, they have been considered for regenerative purposes for Parkinson's disease (PD), multiple sclerosis (MS), and amyotrophic lateral sclerosis (ALS), as well as traumatic brain injury (TBI), spinal cord injury (SCI), or nerve repair [1–3].

Polymers represent an attractive platform for MSCs applications thanks to their tunable properties and high versatility [4–7]. In particular, although their design and optimization set a great challenge, injectable nanocomposite hydrogels (i.e., hydrogels loaded with nanoparticles, NPs) for *in situ* cell or drug release represent a minimally invasive solution to enhance the effectiveness of potential therapeutic strategies and might be usefully exploited for the treatment of severe neurodegenerative disorders like PD and Alzheimer's disease (AD) [4, 5]. In this scenario, coupling MSCs and nanostructured hydrogels might therefore help to take advantage and translate their therapeutic potential into clinical approaches.

In order to strongly support this conclusion, the rheological and functional properties of injectable hydrogels will be preliminarILy discussed; then current applications of nanocomposite hydrogels in MSC-based therapy for nervous tissue applications will be presented, as well as describing the current research carried out by the authors in the field of CNS neurodegenerative disorders, like PD and AD.

## 2. Injectable Hydrogel-Based Nanocomposites: Rheological and Functional Features

Several injectable formulations of gels and hydrogels have been developed for a wide range of biomedical applications [8–16]. In particular, hydrogels have proved to be an optimal tool for cell-based strategies, even though rheological properties of acellular and cell-laden gels might vary considerably [17–19]. From a materials science point of view, rheological and injectability measurements, together with confined compression tests, may provide important information on their functional properties among which the functional injectability plays a crucial role [20].

The injection of a gel could negatively affect its rheological behaviour and viscoelastic properties (storage and loss moduli, G′ and G′′, respectively). In fact, the injection through clinical catheters could cause a partial or total disruption of the polymeric network, thus leading to a decrease of the storage modulus and an alteration of the gel-like behaviour. To improve the viscoelastic properties without altering the gel-like behaviour of both acellular or cell-laden hydrogels [20], NPs are often included as a reinforcement. Accordingly, after the injection, G′ and G′′ may decrease, but the inclusion of NPs may provide values of both dynamic moduli suitable for the specific application [21–25]. The amount of NPs represents a critical factor, since they provide a resistance of the material to flow under shear. The storage modulus and the viscosity normally increase with NP concentration, but beyond a threshold concentration G′ dramatically decreases, even though NP concentration is further increased, and during the injection the NPs act as “weak points” instead of reinforcement. For this reason, the optimization of injectable nanocomposites as well as the prediction of their rheological behaviour represents a great challenge that may be overcome by integrating experimental tests and mathematical models [23]. A very recent example of nanocomposite hydrogels in the specific field of this review is from Koudehi et al. [26]. They developed a nanobioglass conduit for peripheral nerve regeneration by coupling bioglass NPs with gelatin and obtaining a microporous nanocomposite having the pore size of 10–40 *μ*m, good biocompatibility, and good nerve regeneration in a rat model.

With regard to cell-laden nanocomposite gels, small amplitude oscillatory shear tests may be performed at different time points after cell seeding to evaluate G′ and G′′ as a function of frequency, in order to understand the effect of cell behaviour on the viscoelastic properties of the material and to optimize the cell density [20]. At each time point, it is expected that the values of the loss tangent (G′′/G′) are greater than those of the corresponding acellular gels and they increase with culture time.

Viscosity as a function of shear rate may be assessed by steady shear tests; however, the strains induced by oscillatory rheometry are usually smaller than those achieved during extrusion/injection-based measurements and hence less likely to alter the structure of the material [27, 28]. In addition, by performing only small amplitude oscillatory shear tests and steady shear measurements it might be difficult to get shear strains and rates similar to those occurring in clinical practice, where the materials are applied by injection-like methods involving the use of a syringe with a suitable needle. To measure the flow behaviour while simulating the clinical practice, an appropriate injection-based experimental setup is required. For instance, the syringe (equipped with a needle suitable for the final application) may be filled with the material and mounted on a testing machine. By driving the syringe piston at a constant and fixed speed (thus controlling the injection rate and the apparent shear rate in the needle), the material is injected through the needle and the load applied to the piston may be measured by a suitably calibrated load cell. To estimate the friction between the syringe walls and the piston, an empty syringe should be also tested, while to assess the effect of the cells on the flow behaviour over time, the acellular nanocomposite gels should be compared to the cell-laden ones at different time points after seeding. Finally, the results from these injectability measurements may be used to evaluate some functional features and rheological information by applying the basic principles of capillary extrusion rheometry.

Due to the biphasic nature of hydrogel-based materials, confined compression stress-relaxation tests may be also performed on both acellular and cell-laden nanocomposites to evaluate functional parameters, such as the zero-strain compressive modulus, the zero-strain permeability, the nonlinear stiffening coefficient, and the nonlinear permeability coefficient that is a measure of the sensitivity to the deformation. As reported in [27, 28], different equations may be considered to study the case of uniaxial confined compression, starting from the constitutive law for the extra-stress tensor to the relationship between the hydraulic permeability and the axial deformation to obtain a one-dimensional nonlinear partial differential equation.

In terms of transport properties, permeability plays a key role, since the fluid flow through the porous solid matrix controls the deformational process [29–31]. As reported by Ateshian et al. [29] and Williamson et al. [30], the strain-dependent permeability may be properly assessed by fitting the experimental data from the confined compression tests with a nonlinear biphasic model.

In conclusion, all the results from rheological analyses, injectability measurements, and, eventually, transport properties should be taken into account to tailor and optimize the functional features of both acellular and cell-laden gels for tissue engineering applications. Focusing on the CNS, their integration with biological expertise may be fully exploited to pursue innovative approaches involving the synergistic contribution of MSCs and hydrogel-based nanocomposites.

## 3. **Hydrogel and MSCs as a Synergistic Strategy for Nervous Tissue Applications**

Over the past few years, the basic approach of using hydrogels for regenerative or neuroprotective strategies in the nervous tissue has been explored. For instance, Freudenberg et al. [32] developed biohybrid hydrogel platforms based on covalently cross-linked heparin and star-shaped poly(ethylene glycols) (star-PEGs) for potential use in cell replacement therapies for neurodegenerative diseases. They kept the amount of heparin constant and analyzed the effect of the cross-linking degree on the mesh size, swelling, and elastic modulus; furthermore, they demonstrated the impact of biomolecular and mechanical cues on neural stem cells and primary nerve cells.

PEG hydrogels coupled with soluble factors were investigated by Mooney et al. [33], who evidenced how the hydrogel environment may influence neural cell composition and described soluble factors that may be useful in generating neuronal-enriched populations within the specific hydrogel environment.

To obtain successful biomaterial-cell interactions, the modification of synthetic hydrogels with biologically active molecules has become an increasingly important route. For instance, Zhou et al. [34] used the monomer 2-methacryloxyethyl trimethylammonium chloride (MAETAC) to provide tethered neurotransmitter acetylcholine-like functionality with a complete 2-acetoxy-N,N,N-trimethylethanaminium segment. The results from this study showed that MAETAC could promote neuronal cell attachment and differentiation in a concentration-dependent manner.

The expertise gained thanks to the above mentioned scenario paves the way to an intriguing approach: the use of MSCs in cell/material biohybrid constructs for CNS applications. MSCs may be used undifferentiated as a reservoir of trophic factors or they may be differentiated towards a neuronal phenotype for regenerative or replacement therapies. While for the first approach a systemic injection into the blood stream might be suitable [35], for the second one the placement of MSCs at or within the lesion site might benefit from the presence of a hydrogel aiming at ameliorating cell survival and therapeutic performance. For instance, Ghoreishian et al. [36] embedded undifferentiated MSCs from the autogenous adipose tissues of mongrel dogs into an alginate hydrogel and placed the construct into an expanded poly(tetrafluoroethylene) tube to repair a facial nerve lesion. After 12 weeks, an organized neural tissue was formed within the tubes, having a decreased diameter of 29% in the experimental group. Neurofilament-positive axon counts were 67% of normal values and there was no significant difference between the groups in relation to the histomorphometric parameters; furthermore, nerve conduction velocity was significantly greater in the experimental than in the control group. Despite some limitations, these results suggest that a similar approach exploiting different biocompatible materials is promising.

Kubinovà et al. [37] tried a different approach using a methacrylate hydrogel scaffold to support MSC proliferation. They modified poly(2-hydroxyethyl methacrylate) (PHEMA) with cholesterol to get highly superporous hydrogels that may serve as a permissive scaffold for the treatment of spinal cord injury (SCI). *In vitro* the hydrogels supported the adhesion and proliferation of rat MSCs, while *in vivo* they displayed bioadhesive properties and were able to bridge a spinal cord lesion in a rat model. For the same application, other authors [38] proposed an injectable hydrogel with a controlled nanostructure, together with a new protocol for building three-dimensional (3D) biohybrid cell/hydrogel constructs. Their matrix was tested with glial populations, primary astrocytes, and MSCs and the results indicated that the cells survived the period of latency within the hydrogel.

The concept of developing a 3D scaffold to support MSC proliferation or differentiation was also pursued by Gu et al. [39]. They developed macroporous, cellulosic hydrogels to induce the neuronal differentiation of human MSCs (hMSCs). After 7 days the number of hMSCs in the scaffolds increased by more than 14-fold, while after 14 days of induction they could completely differentiate into neurons and glial cells. These results appear very promising, but they might be further improved by the nanostructuration and/or functionalization of the hydrogels. A similar approach was also described by Wang et al. [40]. In this case, the hydrogels were composed of gelatin-hydroxyphenylpropionic acid (Gtn-HPA) conjugate and synthesized by the oxidative coupling of HPA moieties, catalyzed by hydrogen peroxide (H_2_O_2_) and horseradish peroxidase (HRP). Their stiffness was tuned by varying the H_2_O_2_ concentration without changing the amount of polymer precursor. In a 3D context and without any additional biochemical signal, the rate of hMSCs proliferation increased while decreasing the stiffness of the hydrogels. In particular, the cells cultured for 3 weeks in the hydrogel with the lower stiffness expressed much more neuronal markers than those cultured in the stiffer matrices. SCI repair was also attempted in rat models by Hejčl et al. [41] that studied whether it was feasible to bridge a chronic lesion by implanting a hydrogel based on 2-hydroxypropyl methacrylamide functionalized with RGD (Arg-Gly-Asp) sequences either alone or seeded with MSCs. With respect to the control group, the results showed an improvement for the rats implanted with the MSC-laden hydrogels; furthermore, in this case tissue atrophy was prevented, the hydrogels were infiltrated with axons myelinated with Schwann cells, and MSCs were still present in the hydrogels 5 months after implantation. A slight tendency to improvement was also observed for the rats implanted with the hydrogel alone, but it was not statistically significant. Taken together, these results support the therapeutic potential of synergistic approaches integrating MSCs and suitable hydrogels.

A more complex composite material was developed by Park et al. [42], again for the SCI. Their purpose was to test a hyaluronic acid-based hydrogel as a 3D biomimetic scaffold for peptides and growth factors and test this approach *in vivo*. To provide guidance cues for nerve regeneration, they used a matrix metalloproteinase peptide cross-linker, an IKVAV (Ile-Lys-Val-Ala-Val) peptide derived from laminin and brain-derived neurotrophic factor (BDNF). Human MSCs were embedded in the hydrogels and their neuronal differentiation was induced after culturing *in vitro* for 10 days. The hydrogels were then injected *in vivo* into the intrathecal space and the animals were monitored for 6 weeks. If compared to the other tested groups, the rats injected with BDNF-containing materials showed the greatest improvement on locomotive tests during the initial stage after injury.

An important field where NPs might play a key role is MSCs tracking. In fact, the fate of injected or transplanted MSCs into the body in human or animal models is a matter of great debate [43]. To this respect, Zeng et al. [44] investigated the cytotoxicity of superparamagnetic iron oxide nanoparticles (SPIONPs), used as a contrast agent in magnetic resonance imaging (MRI) for labeling cells *in vitro* and tracking labeled cells after transplantation *in vivo*, on the neural differentiation of human amniotic membrane-derived MSCs (hAM-dMSCs). They found that a single SPIONP-labeled method provided appropriate viability for the labeled hAM-dMSCs and facilitated their evaluation by MRI after transplantation. Another similar study investigated the effective concentration of SPIONPs to track MSCs by evaluating the influence and toxicity of their labeling on multiple differentiated MSCs. The results indicated that the cells were effectively labeled at low concentrations of SPIO and in this condition they maintained their proliferative features and differentiation capacity [45].

## 4. MSCs in Parkinson's and Alzheimer's Diseases

Several data have already outlined that MSCs might play a role in AD/PD regenerative or neuroprotective approaches. In fact, MSCs produce a variable panel of protective soluble factors that might be released from a nanostructured hydrogel to the brain parenchyma. For instance, a recent paper by Lee et al. [46] indicated that bone marrow (BM) stem cells showed a beneficial effect through endogenous microglia activation in the brains of AD mice thanks to the selective production of the chemoattractant factor CCL5. Another group reported that soluble intracellular adhesion molecule-1, secreted by human umbilical cord blood-derived MSCs, reduced amyloid-*β* plaques both *in vitro* and *in vivo*, probably thanks to the activation of an amyloid degrading enzyme [47].

In the field of PD, Whone et al. [48] investigated the paracrine effect of MSCs, focusing on the release of glial-derived neurotrophic factor (GDNF). They investigated the neuroprotective properties of unmodified hMSCs on rat catecholaminergic and serotonergic cell cultures exposed to nitric oxide. They found that the soluble factors produced by native hMSCs, requiring no direct cell-cell contact, gave protection not only to cultured monoaminergic perikarya, but also to monoamine neurotransmitter transporter function, and that these beneficial effects were mediated in part by GDNF release.

**Figure 1:**
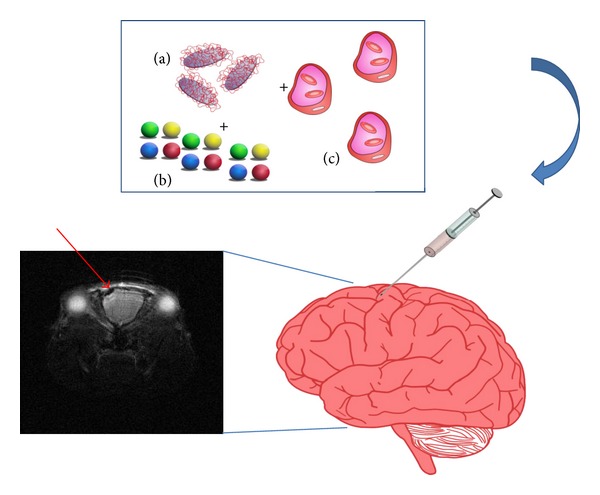
Schematic summarizing a possible application of nanostructured gels in the brain. The nanostructured device, composed of (a) an injectable, biocompatible, and resorbable hydrogel, is mixed with a nontoxic amount of NPs (b) and loaded with MSCs (c) that are able to naturally phagocyte the NPs. The device is injected into its target site and the resulting labeled MSCs might be imaged by MRI or other noninvasive techniques. The insert shows an MRI scan of a mouse brain, where the dark spot indicated by the arrow is a denser signal due to SPIONPs.

All these data support the research in the field of nanocomposites and their application for complex disorders like AD/PD. In this field, the main challenges are the target tissue (brain) that is hardly accessible and the chronic feature of the disorders, requiring prolonged therapies over time (Figure 1).

## 5. Conclusions and Perspectives

In summary, several hydrogel formulations have been specifically developed to mimic 3D scaffolds or nanostructured devices for the release of trophic factors or peptides favoring MSCs adhesion, differentiation into the neuronal lineage, and survival. Considering that NPs are also very promising tools for MSC tracking and taking advantage from the natural phagocytic activity of MSCs, an easy working hypothesis might be the development of a composite gel, nanostructured to label the released or embedded MSCs. This approach might increase the labeling efficiency and decrease possible toxic effects mediated by the NPs, thanks to the copresence of trophic factors. A general comment is that it is mandatory to improve surgical and delivery strategies, as the nervous tissue, and the brain tissue in particular, sets problems of reachability without relevant side effects (unwanted neuronal damage and neuroinflammation). In this field, recent works have mainly focused on SCI, a peripheral and thus more easily addressable pathology from the point of view of hydrogel-based therapies. To this end, a recently described epicortical approach for the release of drugs to the brain after a stroke might also be promising for drug delivery [49].

**Figure 2:**
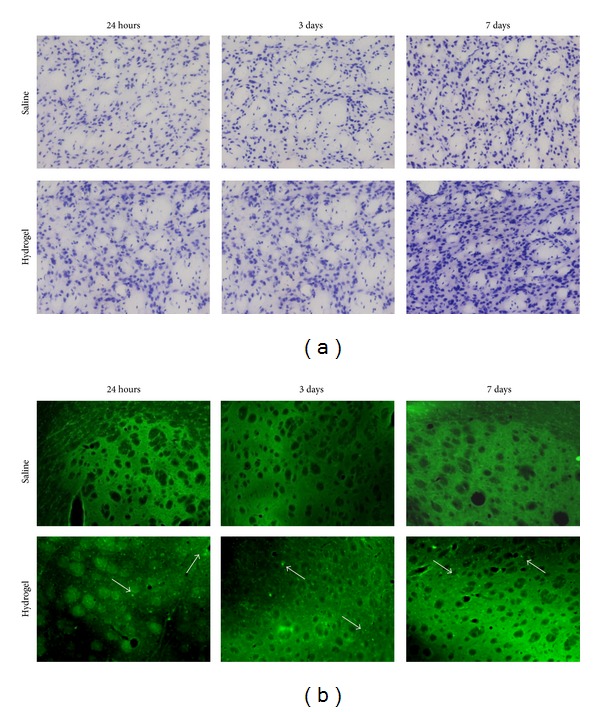
Evaluation of the biocompatibility of an injectable hydrogel formulation for brain applications. We designed and prepared in saline (0.9% NaCl) a hydrogel (pH 7.2) composed of Carbomer 974P (4.3% w/v), PEG (Mw = 600 g mol^−1^, 12.2% v/v), and glycerol (14.6% v/v). An aliquot (2 *μ*L) was diluted 1 : 1 (v/v) with saline to simulate the cell loading and stereotaxically injected into the mouse striatum, keeping the contralateral brain hemisphere as a control. (a) Nissl staining to point out brain tissue cytoarchitecture. In the hydrogel-injected hemisphere, tissue hypertrophy was present after 7 days. (b) Fluoro-Jade A staining. This staining allows tracking cell damage points that appear as bright green dots. Differently from the control, where few dots were visible, in the hydrogel-injected hemisphere neuronal and glial damage was detectable as early as 24 h after injection. The white arrows point to the Fluoro-Jade A positive points.

The authors of this review are currently working on the nanostructuring of semi-interpenetrating polymer networks based on collagen and PEG, with the specific focus of their optimization for brain delivery that, as outlined, is a critical and unmet improvement for advanced therapies based on biocompatible devices. In particular, we are investigating the effects of NPs in modulating the rheological and functional properties of the hydrogels and also improving the sustained release of therapeutic recombinant proteins (i.e., the chaperone protein Hsp70 that has proved to be beneficial in several models of neurodegeneration) [50–53]. Our view is that nanocomposite gels might be a powerful tool to achieve a controlled and long-lasting drug delivery in close contact with the damaged areas in the case of chronic neurodegeneration, an innovative strategy that might open the way to new therapies [54]. To this purpose, the development of highly biocompatible hydrogel-based matrices is mandatory to avoid unwanted tissue damage or trigger a dangerous inflammatory response in the very peculiar biologic environment represented by the brain nervous tissue. To clearly demonstrate the latter point, in Figure 2 we show a time course of a biocompatibility study to assess the brain cytoarchitecture and inflammatory response after the injection of a hydrogel composed of Carbomer 974P (2.15% w/v), PEG (Mw = 600 g mol^−1^, 6.1% v/v), and glycerol (7.3% v/v) and prepared in saline (pH 7.2). Just after 24 h, we noticed tissue damage and neurodegeneration, while after 7 days hypertrophy and neurodegeneration were apparent in the hydrogel-injected hemisphere, suggestive of a negative response to the biomaterial, even though this formulation had suitable rheologic and structural properties. From this example it is apparent that our work, with its focus on brain biocompatibility, is an original step forward in the field.

**Figure 3:**
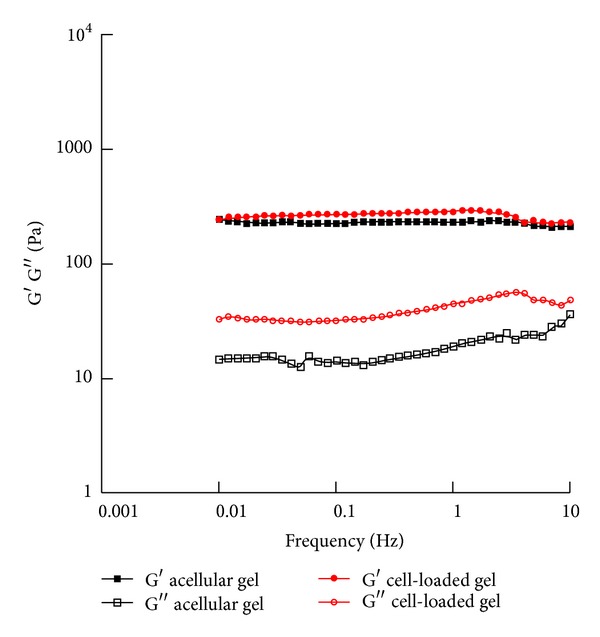
Small amplitude oscillatory shear tests. After the preparation, small amplitude oscillatory shear tests were performed on acellular and cell-laden (0.5·10^6^ cells/mL gel) PEG/Carbomer-based hydrogels at 37°C with frequency ranging from 0.01 to 10 Hz. Both acellular and cell-laden materials showed a gel-like behaviour as the storage modulus G′ was greater than the loss modulus G′′ in the frequency range investigated. In particular, G′ was almost constant for both acellular gels (227.5 ± 34.1 Pa) and cell-laden constructs (280.0 ± 41.0 Pa).

**Figure 4:**
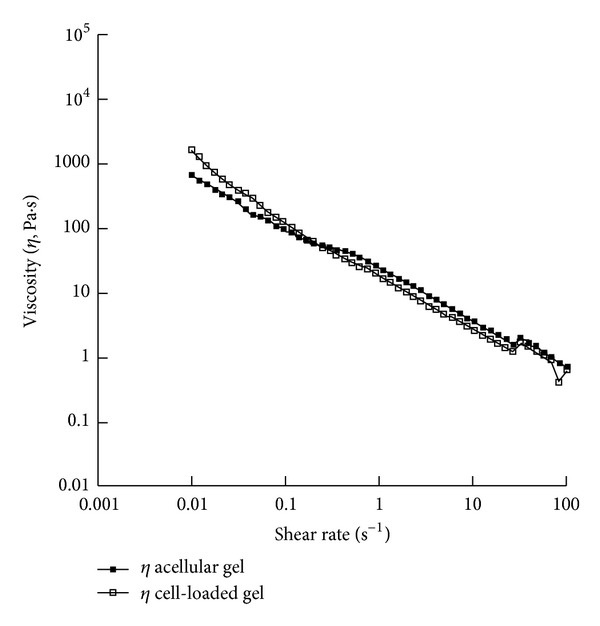
Steady shear tests. After the preparation, steady shear measurements were performed on acellular and cell-laden (0.5·10^6^ cells/mL gel) PEG/Carbomer-based hydrogels at 37°C with shear rate ranging from 0.01 to 100 s^−1^. In both cases, the viscosity decreased as the shear rate increased (shear thinning behaviour). In particular, when the shear rate increased from 0.01 to 100 s^−1^, the viscosity decreased from 664.5 to 0.7 Pa·s for acellular gels and from 1608.1 to 0.6 Pa·s for cell-laden constructs.

As highlighted in the first section, viscoelastic features, functional injectability, and transport properties (i.e., permeability), together with biocompatibility and biological response, play a crucial role in driving the performances of hydrogel-based devices. In particular, the embedded cells may strongly change the properties of the starting material and we aimed at assessing this point focusing on MSCs embedding. As an example to clearly evidence this feature, in Figures 3 and 4 we show the results from small amplitude oscillatory shear tests and steady shear measurements for the acellular and cell-laden PEG/Carbomer-based hydrogels. The results indicated that the presence of the cells initially increased the stiffness (as suggested by the fact that G′ was lower for acellular materials) and the resistance to flow under shear, especially at low shear rates (as shown by the higher viscosity values for cell-laden constructs).

However, to gain a deeper knowledge about the effects of cell loading on the hydrogel properties, these features should be assessed at different time points after cell seeding. Moreover, the evaluation of how the injection through clinical catheters (already applied for Parkinson's disease MSC-based experimental therapies [55]) modifies the viscoelastic properties might represent the first step of a systematic analysis for hydrogels and nanocomposites optimization in the view of a clinical application.
